# Cluster-randomized, controlled evaluation of a teacher led multi factorial school based back education program for 10 to 12-year old children

**DOI:** 10.1186/s12887-018-1280-y

**Published:** 2018-09-26

**Authors:** Silvia Dullien, Joachim Grifka, Petra Jansen

**Affiliations:** 1 Department of Orthopaedics Regensburg University Medical Centre, Asklepios Klinikum, Bad Abbach, Germany; 2 Department of Sport Science, University of Regensburg, Universitätsstr. 21, 93053 Regensburg, Germany

**Keywords:** Back pain, Prevention, Children, Back education, Controlled

## Abstract

**Background:**

The aim of this cluster-randomised, controlled study was to examine whether a teacher-led multifactorial back education programme could improve back pain in pupils, motor skills, back behaviour, and back knowledge over a 10-month period.

**Methods:**

There were 176 children from two schools, who were cluster-randomised into intervention and control groups. The intervention programme consisted of 3 parts: 1) knowledge improvement, 2) posture awareness training, and 3) reducing imbalance of core muscles through mandatory back and abdominal muscle exercises at the beginning of each physical education lesson. Outcome measures included a clinical orthopaedic examination, a health questionnaire, a motor test, a back-behaviour trial, and a knowledge test.

**Results:**

Clinical examination showed a reduction of orthopaedic abnormalities in both groups, from 90.5 to 42%, with a posture test showing an improvement in both groups at the post-test. However, the rate of children reporting back pain at least once a month could not be reduced below 30%. Long lasting physical activity, carrying heavy schoolbags, and long periods of sitting were the top three causes for back pain. Push-up number and balancing skills improved significantly in both groups from pre- to post-test. In the water crate carrying task and knowledge test, only the intervention group (IG) showed a statistically significant improvement from pre- to post-test.

**Conclusions:**

The results show that back pain rate could not be decreased. However, back care knowledge and parts of back-friendly behaviour could be significantly improved. On the other hand, the problem of prolonged sitting and using heavy schoolbags persists.

**Trial registration:**

Deutsches Register Klinische Studien DRKS00013794; Date of Registration: 15.1.2018; Retrospectively registered.

**Electronic supplementary material:**

The online version of this article (10.1186/s12887-018-1280-y) contains supplementary material, which is available to authorized users.

## Background

Back pain is globally the most frequent cause of disability. In 2015, nearly 540 million people suffered from it [1]. When entering the school system, back pain becomes a subject for children and adolescents. From there, the prevalence rates increase until they reach adult rates at the age of 18 [2]. It is similarly known that back pain in the younger years is associated with back pain as an adult [3]. Risk factors for back pain in children are, among others, poor overall fitness, heavy work in leisure time, reduced quality of life (QoL) [4], higher body mass index (BMI) [5], trunk asymmetry in girls [6], asymmetric carrying of the school bag [7], schoolbag carrying time [8], and physical inactivity [9].

School-based intervention programmes tackling these risk factors are necessary, having already been conducted in many countries [10–15]. So far, no school-based study has been conducted in Germany, as the programme was led by a teacher; it provides an advantage, in that every school can establish these interventions on their own.

## Methods

### Objective

Another aim of this study was to examine if teacher-led intervention programmes could improve back-care knowledge, back-friendly behaviour, and core muscle endurance in pupils.

### Participants

The intervention programme was tested on 10- to 12-year-old pupils in the 5th grade at two German “Gymnasiums.” Four classes from each school participated. However, in a school environment, individual randomisation was not possible, such that whole classes were cluster-randomised. Two classes per school were randomly assigned to the intervention group (IG) and two classes were assigned to the control group (CG), resulting in four intervention classes and four control classes. For the cluster-randomisation, the authors chose a class for the respective condition by lot. The children in the IG were asked not to talk about the programme, as the poster for the respective exercises were only in the classrooms of the IG children. Yet carry-over effect could not be excluded, as children might have talked in the schoolyard about it. All participants were required to bring a written informed consent from their parents. At the beginning of the school year, a total of 176 children took part in the baseline assessment (100 girls/76 boys).

During the pre-test, the IG consisted of 90 children and the CG consisted of 86. Due to missing questionnaires, pupils moving away or feeling ill on one of the measurement days, resulted in the actual number of valid cases (varying between three measurement points). The mean age of the IG at pre-test was 10.6 years (± 0.44) (CG 10.5 (± 0.43)), the mean height of the IG was 1.45 m (± 0.75) (CG 1.45 m (± 0.066)), and the mean weight of the IG was 37.5 kg (± 8.10) (CG 38.0 (± 7.26)). None of the above-mentioned parameters showed significant differences. Furthermore, there were no differences in the appearance of trunk asymmetries, which is seen in scoliosis or hollow back (both *p* > 0.6) (Table 1).

**Table 1:** Anthropometric data of study participants at baseline measurement, (Mean, standard deviation and *p*-value separated by group at baseline)

	Group	N	Mean	SD	*p*-values, t-test, Chi-squared-test
Age (years)	Intervention	87	10.59	.438	.296
	Control	85	10.52	.426	
Height (m)	Intervention	90	1.44	.074	.710
	Control	86	1.45	.065	
Body weight (kg)	Intervention	90	37.49	8.10	.640
	Control	86	38.04	7.26	
Body-mass-index (kg/m2)	Intervention	90	17.71	2.40	.718
	Control	86	17.84	2.50	
Performance Matthias-test (sec)	Intervention	90	48.61	14.84	.058
	Control	87	52.82	14.51	
Amount of physical activity ^1^	Intervention	78			.655
	Control	72			
Status of the back muscles^2^	Intervention	89			.313
	Control	84			
Status of the Spine^3^	Intervention	85			.637
	Control	77			

^1^0 = never, 1 = sometimes, 2 = once a week, 3 = twice a week

^2^1 = good, 2 = inconspicuous, 3 = lankly

^3^1 = inconspicuous, 2 = conspicuous

### Interventions

The intervention programme consisted of three parts: 1. Knowledge improvement through five lessons on back care, which was held by a teacher with the provided material, 2. Posture awareness training and improvement in the classroom with three posters, and 3. Reducing muscular imbalance of the core muscles through mandatory back and abdominal muscle exercises at the beginning of each lesson.

**Fig. 1:**
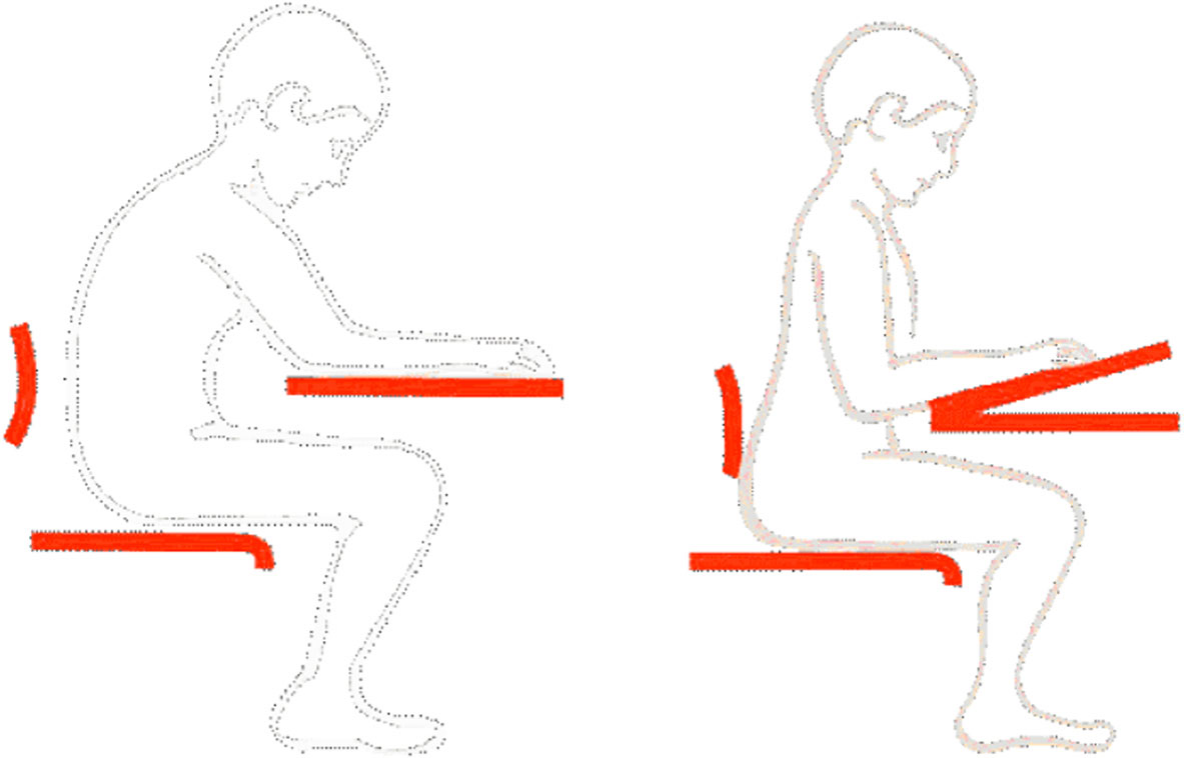
Good (left side) and bad posture (right side) while sitting

The five lessons were developed in cooperation with orthopaedic residents, psychologists, sports scientists, and teachers. They focused on anatomical knowledge of the back and spine, good and bad posture while sitting (see Fig. 1), healthy backpack habits, healthy lifting and carrying, and back-friendly sports and nutrition. To promote good posture, it was explained that dynamic sitting involved changed positions as being relevant. Healthy lifting and carrying was explained by examples such as correct lifting through bending the knees for the consistent distribution of weight, etc. Back-friendly sports like swimming and skating were introduced, as well as the importance to reduce sitting behaviour.

Posture awareness training consisted of three posters mounted in the classroom. The first poster showed alternative sitting variations to promote dynamic sitting. The second poster showed strengthening exercises for the core muscles. The third poster showed mobilisation/stretching exercises to improve muscular tensions and shortenings. All of these exercises could be performed at the pupils’ desks. At least one of the stretching and one of the strengthening exercises had to be performed at the beginning of a school day and at another time chosen by the teacher, on an individual basis. The teachers had been given calendars to note how often the posture awareness training was administered per day. However, most teachers did not write down the number of these exercises as the study period progressed. So, this data was not complete and thus, has not been considered for data analysis. For the mandatory back and abdominal muscle training, every participating physical education teacher received an exercise collection with a detailed description of every exercise. There were static and dynamic exercises. The static exercises should be completed three times, with each position held for 15–20 s. For the dynamic exercises, each one should be conducted with 15–20 repetitions. All exercises were explained in written form, as well as with a photo showing its correct execution. Examples of the exercises involved: plank, crunch, hip lifts, flexion of the back muscles, and ball-exercises.

### Outcome measures

Pupils had to complete several different tests (see Table 2). They had to complete motor testing, a back-behaviour trial, a clinical exam with an orthopaedic surgeon, a health questionnaire, and a back-related knowledge test. These outcome measures (see Additional file 1) were fulfilled by the children two times (pre-test and post-test). In only the intervention classes, there was an additional mid-term evaluation (after completing the five back-care education lessons), in which only the back-behaviour trial and the back-related knowledge test were carried out.

**Table 2:** Measurement time points and outcome measures

Measurement points	Test instruments	Outcome measure	Details/Source
1. Pre-test At the beginning of the school year Intervention-and Control-Group	1. Clinical orthopaedic examination	Body weight, body height Orthopaedic abnormalities of the spine Posture Test: Matthiass-Test	
	2. Health questionnaire	Anamnestic questions How often do you have back pain?	Descriptive Data
	3. Motor Tests	Push-ups Sit-ups Balance test Stand and Reach Hanging on wall bars	Posture Test for children [16] see above see above see above Munich fitness test [17]
	4. Back-behaviour Trial	Back pack handling Demonstrate sitting postures Demonstrate strengthening exercises Carrying a water crate	
	5. Knowledge Test	12 questions on healthy back knowledge	
2. Mid-term evaluation After 4 months Intervention group only	Back-behaviour Trial Knowledge Test		
3. Post-test At the end of the school year Intervention- and Control-Group	1. Clinical orthopaedic examination 2. Health questionnaire 3. Motor Tests 4. Back-behaviour Trial 5. Knowledge Test		

Each child who participated was examined with an orthopaedic resident. Body height, body weight, and abnormalities of the spine were noted, while asymmetries of the upper body were checked and categorised for the back in normal, flat, hyperkyphotic, or hyperlordotic positions. The health questionnaire asked anamnestic questions about overall health and immune-driven responses, to diagnose back pain/disorders and its frequency.

To examine children’s motor skills, four muscle endurance tests involving core muscles were used. The number of push-ups and sit-ups the children were able to complete were measured as well. The respective number was registered. Furthermore, while balancing on one leg on a T-shaped bar, floor contacts with the opposite leg during the 40 s were noted to assess postural control and balancing skills. Lastly, it was measured how far they could reach their arms onto the ground while standing and not bending the knees. The tests are described in Ref. [16]. The test “Holding onto wall bars” quantifies upper body muscle endurance. The children were asked to hold onto the uppermost bar of a wall bar without the foot contacting for as long as possible. At the moment, the nose tip fell under the bar level, counting seconds stopped. This test is a part of the “Münchner Fitness-Test” [17].

The back-behaviour trial consisted of four tasks. Task 1 was lifting, carrying, balancing on a marked line, correct turning, and putting down a mineral water crate. For each of the tests in this task, 0–2 points could be achieved. For example, for the lifting task, the children received 0 points if they lifted the crate with set-through knees, 1 point for bent knees and bent back, and 2 points for the correct execution. Task 2 was packing a backpack correctly, not exceeding individual weight limit, correct positioning, carried on both shoulders, and adjusted on the back correctly. It was registered if the children could complete the task correctly (2 points), partly correctly (1 point), or not at all (0 point). Concerning task 3, children were asked to demonstrate four different sitting positions which could improve postural dynamism. For every correct sitting position, 1 point was received. For task 4, pupils were asked to demonstrate two strengthening exercises for the abdominals and back muscles, with one flexibility-exercise. They received 2 points if the exercise was completely executed, 1 point if there were small anomalies, and 0 points if they were not completed correctly. Forty points were available. The knowledge test consisted of 12 questions, which related to five back-care lessons: in total, there were 24 points. The orthopaedic resident and tester were blinded.

### Statistical methods

All analyses were performed using SPSS 18 (IBM Inc., Chicago, IL, USA). The level of significance was set at *p* < 0.05. For motor testing, as well as the back-behaviour trial, a univariate analysis of variance was calculated with the factor “test time” (pre-test, post-test), and the factor “group” (IG vs. CG). A possible difference of the back-pain rate between groups was calculated with the Wilcoxon-Test.

## Results

In the following, only meaningful or statistically and clinically significant results are shown.

### Orthopaedic abnormalities of the spine

For the pre-test, 162 children had been examined clinically, indicating that 10.5% had a clinically unremarkable spine. At post-test, 58% had a clinically unremarkable spine. The rate of improvement was equally distributed between the IG and CG. A possible scoliotic deformity was noted in 1.9% (3 cases, 1 boy and 2 girls) at pre-test. Six children (3.7%) had hyperkyphosis at pre-test. At the post-test, this rate was reduced to 3 cases (1.7%).

### Posture test “Matthiass-test”

**Fig. 2:**
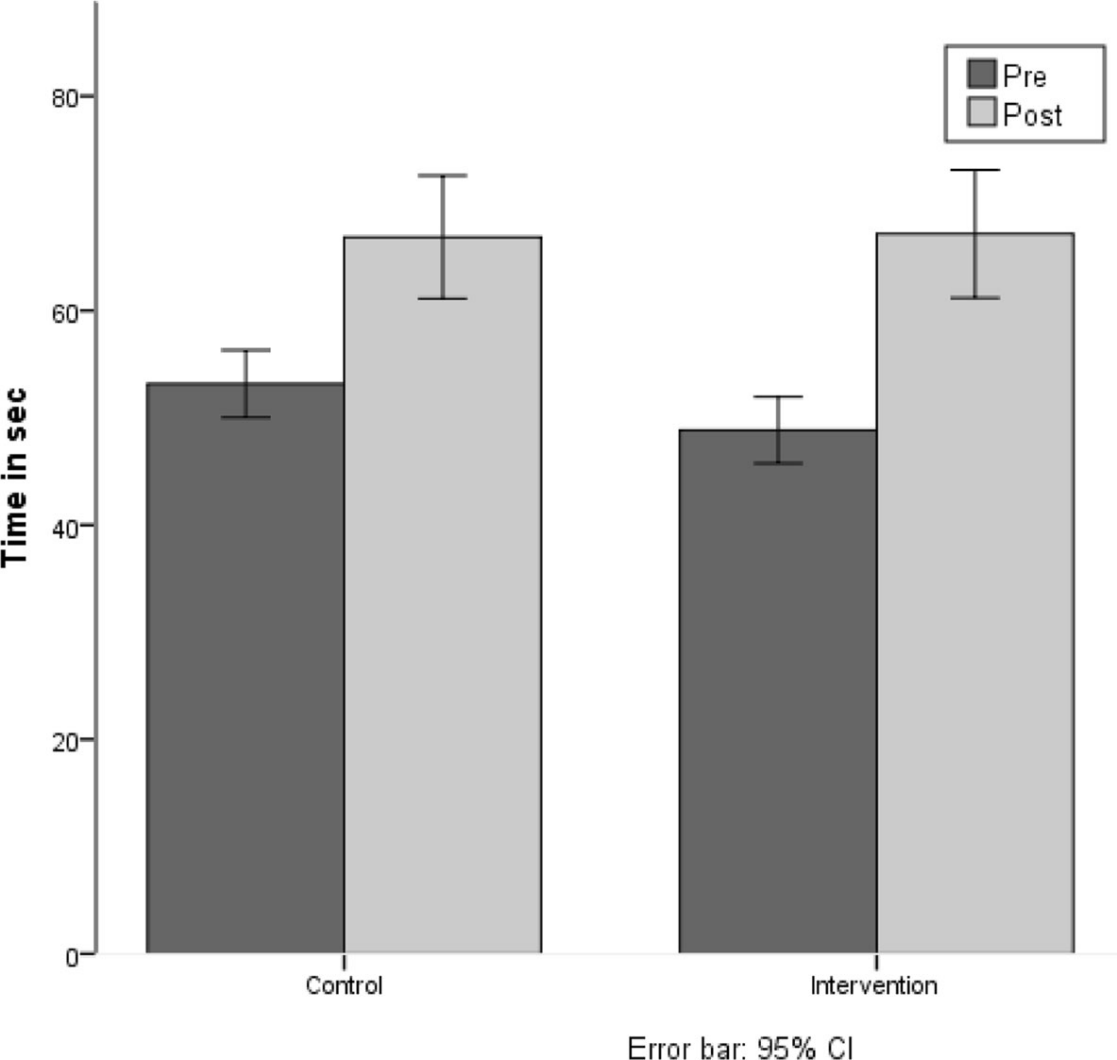
Time in sec posture could be held correctly in the posture test

The results of the posture test (Matthiass-Test) show that the IG as well as the CG improved their performance between the pre- to post-test. There was no difference in the training gain of the IG compared to the CG, (F(1,171) = 1.02, n.s.) (see Fig. 2).

### Back pain frequency

**Fig. 3:**
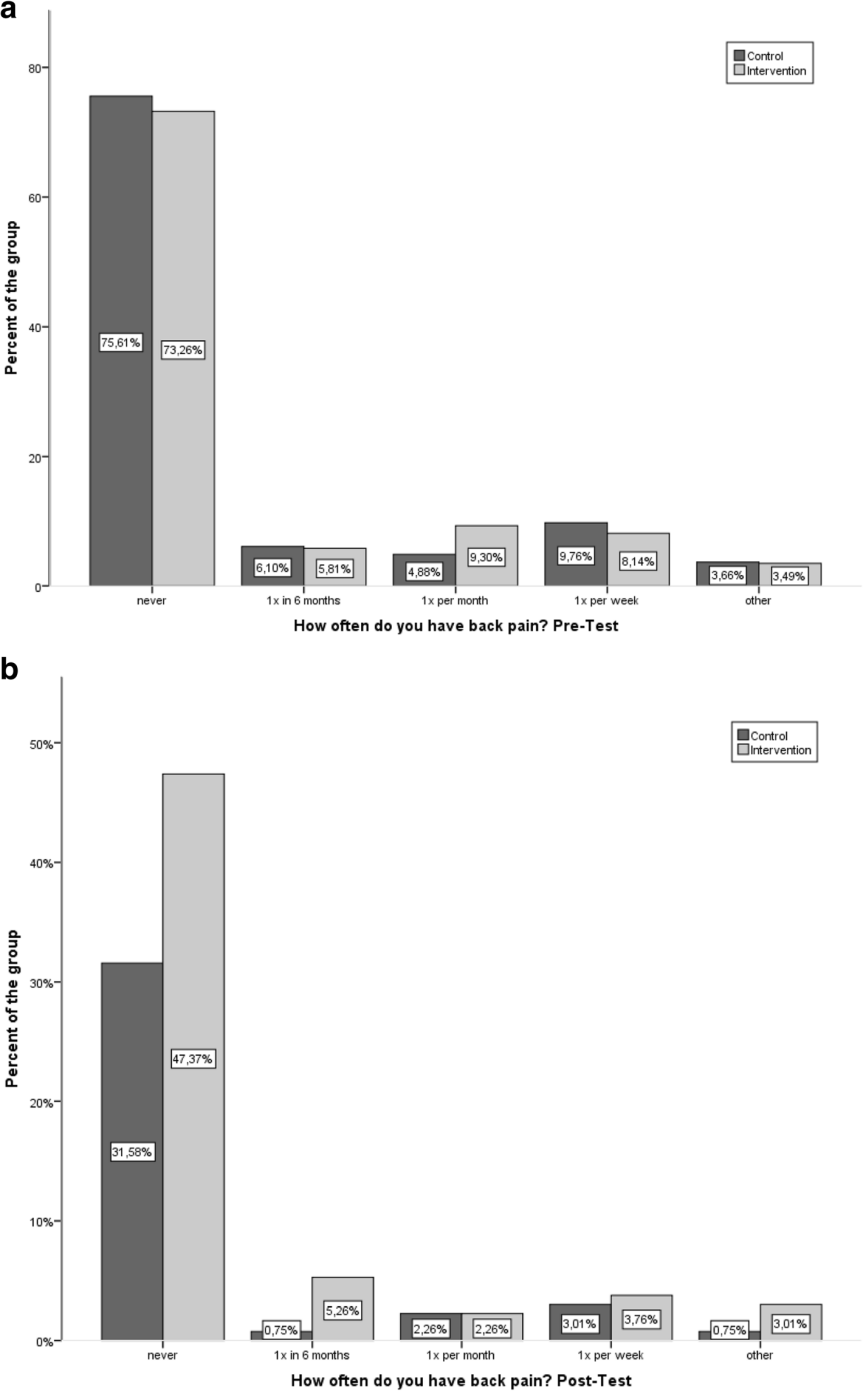
Frequency of back pain at pre-test (Fig. 3a) and post-test (Fig. 3b) of all children divided into IG and CG

Of 168 children who submitted the health questionnaire at pre-test, 125 (68.7%) did not suffer back pain – but 15 children (9%) stated that they did suffer with back pain once a week. Thus, over 30% of the children had back pain, with the most frequent cause being long-lasting physical activity: a long hike (*n* = 10), carrying a heavy schoolbag, and long periods of sitting (both *n* = 9). Of the 43 children with back pain, there were 22 boys and 21 girls. The chronological breakdown for the pre-test is shown in Fig. 3, differentiating IG and CG. Comparing the post-test back pain rates, there was neither a reduction of back pain frequency, nor were there significant differences between the groups (Z = − 0.203, *p* = 0.839).

### Core-muscle endurance tests

#### Push-ups

**Fig. 4:**
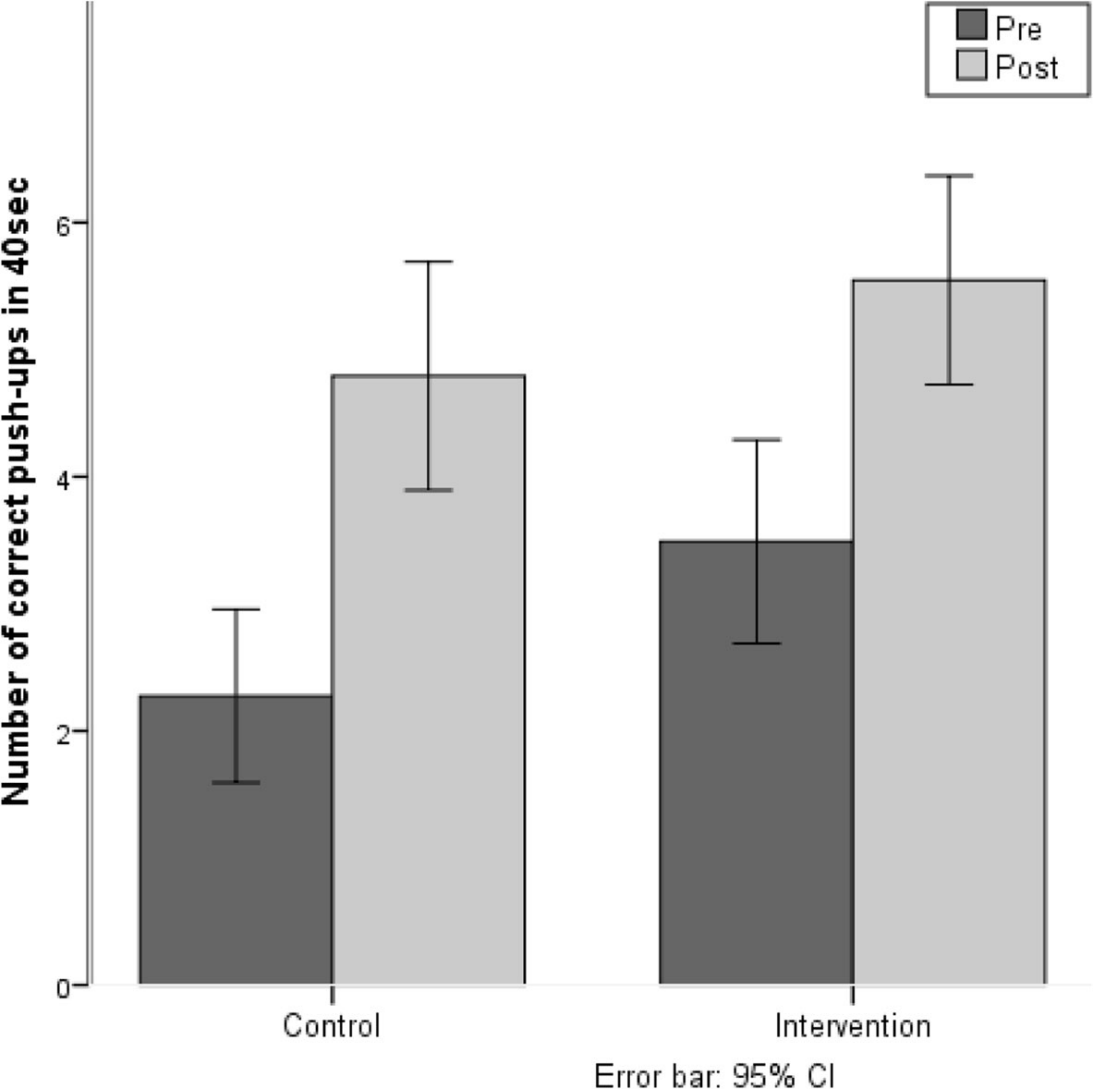
Number of correct push-ups in 40 s’ pre-test and post-test

At pre-test, the mean number of push-ups for the IG was 3.4 (± 3.8) and for the CG it was 2.2 (± 3.0). At post-test, the mean number of push-ups for the IG was 5.6 (± 3.9) and for the CG 4.9 (± 4.0). The results show that the intervention, including the control group, showed better performance from the pre- to post-test (F(1,163) = 80.76, *p* < 0.001.) There was no difference in the training gains of the IG compared to the CG (see Fig. 4).

#### Balancing on a T-bar

**Fig. 5:**
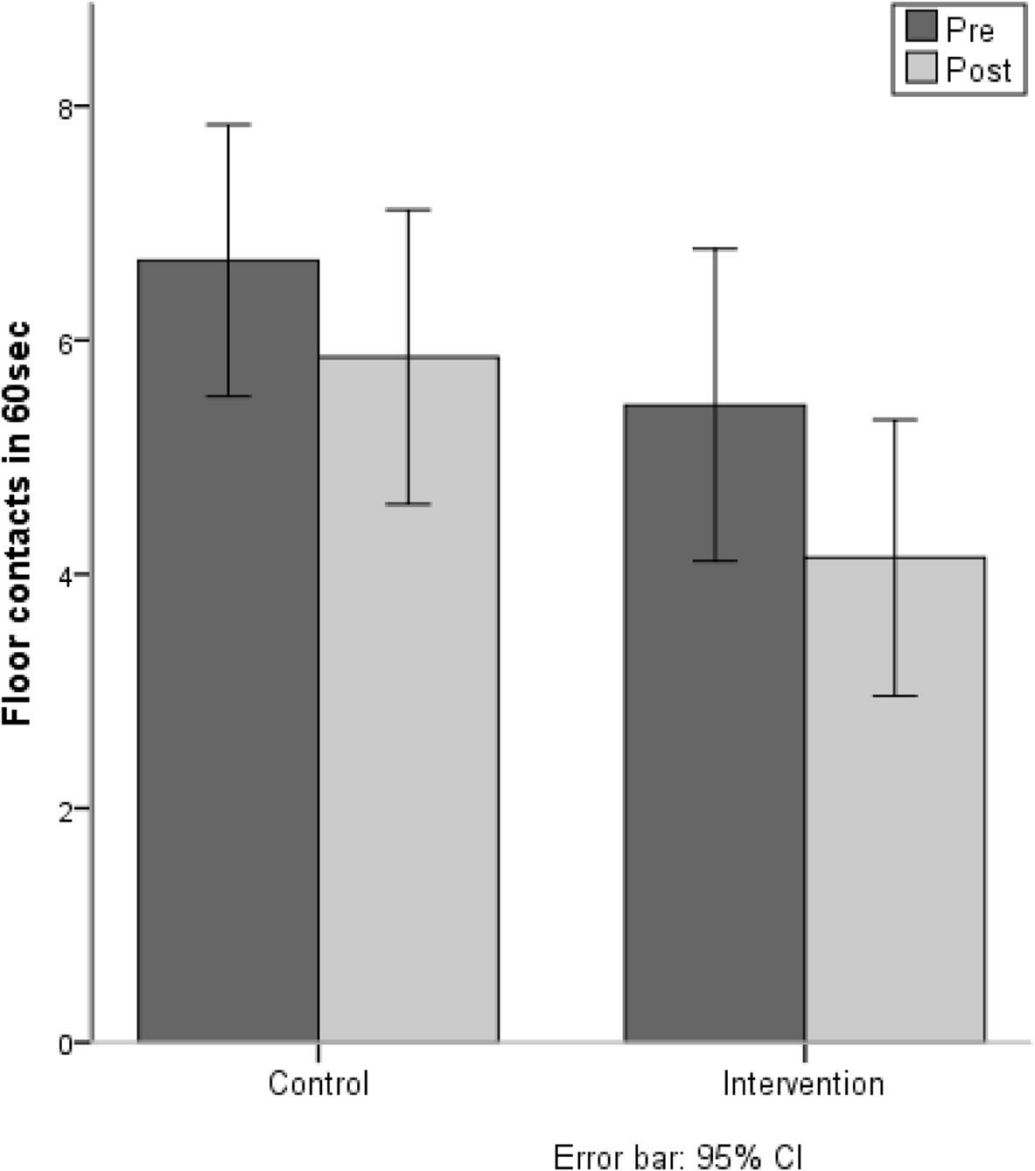
Number of floor contacts in 60 s balancing on one leg on a t-shaped bar

At pre-test, the mean number of floor contacts for the IG was 5.4 (± 5.0) and for the CG it was 7.1 (± 4.8). At post-test, the mean number of floor contacts for the IG was 4.9 (± 4.7) and for the CG 6.6 (± 5.3). These results show that the IG as well as the CG showed better performance from the pre- to the post-test (F(1,117) = 6.76, *p* < 0.05). There was no difference between the trainings gain of the IG compared to the CG (F(1,171) = 0.341, n.s.), (see Fig. 5).

#### Sit-ups

At pre-test, the mean number of sit-ups for the IG was 20.52 (± 4.55) and for the CG 18.29 (± 4.42). At post-test, the mean number of sit-ups for the IG was 20.00 (± 4.89) and for the CG 19.64 (± 4.69). The results show no significant main effect of time, (F(1,116) = 1.56, *p* = 0.242), but of group, (F(1,165) = 4.097, *p* = 0.045) and a significant interaction between both factors, (F(1,165) = 7.92, *p* = 0.005). Only a difference between groups was seen in the pre-test.

#### Stand-and-reach

Concerning the stand-reach performance, there was no main effect of time, (F(1.165) = 0.114, *p* = 0.737, nor group, (F(1.165) = 0.005, *p* = 0.944), nor an interaction between both factors, (F(1,165) = 0.804, *p* = 0.371).

#### Carrying a water crate

**Fig. 6:**
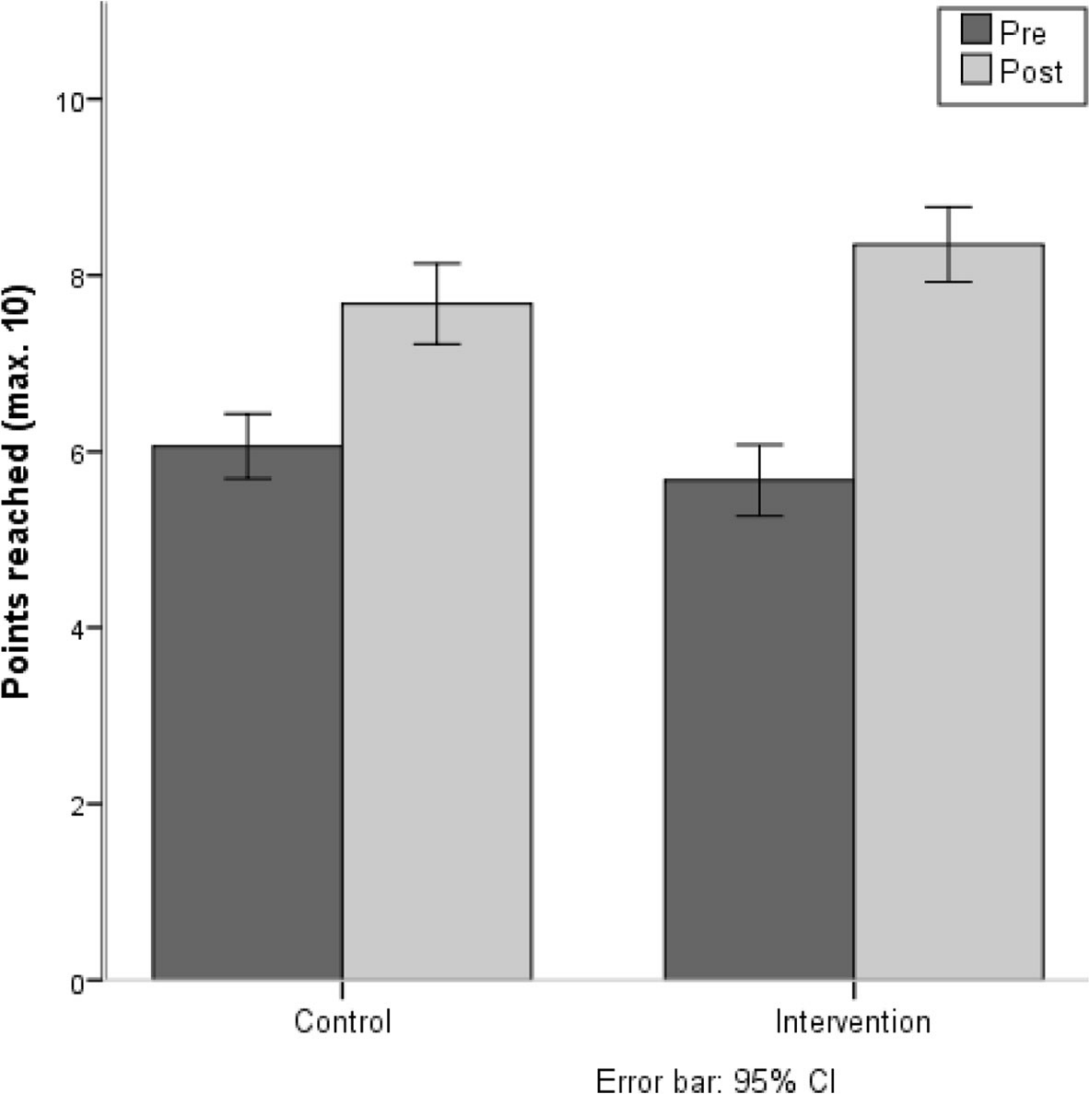
Points in the “water crate-carrying task”

At pre-test, the mean number of points in the “water crate task” for the IG was 5.7 (± 1.9) and for the CG 6.1 (± 1.7). At mid-term evaluation, the IG mean result was 7.71 (± 2.1) and at post-test, the mean number of points for the IG was 8.2 (± 2.0) and for the CG 7.7 (± 2.1). Results show a significant interaction between the factors “group” and “time of testing” (F (1.164) = 7.93, *p* = 0.005). Only the IG improved their behaviour from pre- to post-test (see Fig. 6).

### Knowledge test

**Fig. 7:**
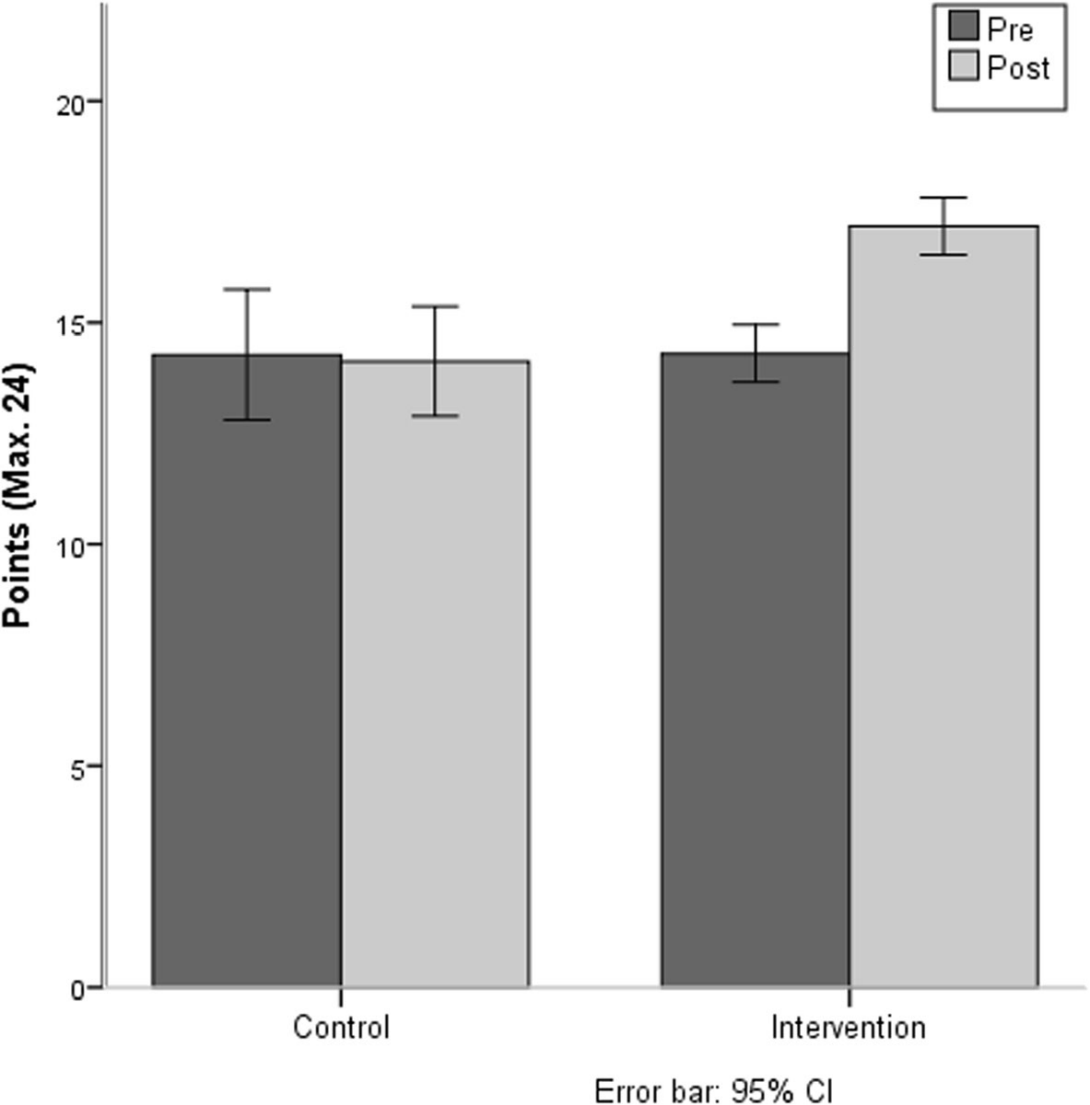
Points received in the knowledge test

At pre-test, the mean number of points in the “knowledge test” for the IG was 14.42 (± 3.03) and for the CG 14.80 (± 5.05). At mid-term evaluation, the IG mean result was 16.8 (± 3.76) and at post-test, the mean number of points was 17.17 (± 2.84), and for the CG 14.57 (± 4.42). Concerning the answers to the subject of “back education measured in points received in the knowledge test”, there was a significant interaction between the factors “group” and “test time” (F (1.123) = 11.87, *p* = 0.001). The result indicates that only the IG significantly improved their knowledge from the pre- and post-test, see Fig. 7.

## Discussion

The aim of this study was to examine if a teacher-led intervention programme could improve back-care knowledge, back-friendly behaviour, and core muscle endurance.

Our results indicate that the rate of clinically unremarkable spines augmented enormously from pre- to post-test. The blinded experienced examiner (one orthopaedic resident with expertise in paediatric orthopaedics and experience in diagnosing and treating scoliosis) remained the same both times. In both groups, body height increased from pre- to post-test (CG 3 cm and IG 4.5 cm). Perhaps this is why some orthopaedic abnormalities disappear with time due to natural body development over the 10-month study period. The identified scoliosis rate of 1.9% is in accordance with the 2% found in the literature [18].

The rate of back pain (30%) is comparable to reported prevalence rates. The mean lifetime prevalence in children as seen in a meta-analysis across 30 studies by Calvo-Munoz was 0.399 (95% CI: 0.342 and 0.459) [19]. The leading three causes of back pain are physical overload situations, so the focus must be on back pain prevention programmes.

The results of the motor tests (push-ups and balancing) showed improvements for the IG as well as the CG. One reason could be the learning effect, which appeared when involved in the tests for the second time. The conclusion could be that the quality of physical education lessons in the two schools was already good and following the guidelines of the intervention programme. A German gymnasium in a rural area, where children are often organised in sports clubs, might have fitter children than normal. Might the programme have a greater effect in a disadvantaged urban area school, with a high percentage of migration backgrounds.

In the back-behaviour trial, only the water crate-carrying task showed a significant difference at post-test between groups. It is possible that the pupils of the IG talked about the programme in the schoolyard, so that a carry-over effect occurred in other tasks. The better performance of the IG in the water crate-carrying task is crucial, because healthy lifting habits are important for development of healthy back behaviours in childhood and youth. A behavioural education, which starts at a young age will have a more profound and lasting effect during one’s life. In the knowledge test, the IG received statistically significant more points in the post-test than the CG. According to this, it can be stated that the programme informed the IG of some relevant knowledge, which coincides with a former systematic review [10]. It supports the results of Gelhof et al. [11], in that a one-year teacher led programme and not only a two-year programme were able to improve the children’s knowledge significantly. In addition to the study of Vidal et al. [14], this study has the advantage that the effects of knowledge about the spine, healthy behaviour, as well as physical changes are consistently investigated. Kamper, Yamato, and Williams analysed all systematic reviews for possible reasons in childhood [20] and concluded that the studies differ widely due to their quality (low quality, moderate quality, or high quality). They indicated that psychosocial stress as well as other psychosocial factors reinforce the risk of back pain. Girls have a higher risk than boys do, which should be considered in planning further intervention programmes.

## Conclusion

The results show that back-care knowledge, and aspects of back-friendly behaviour were significantly improved through the programme. There was no significantly improved behaviour concerning core muscle endurance, tested with the four muscle tests mentioned above (between the IG and CG). Since motor results did not improve as well as knowledge and behavioural tests, physical education in the schools was likely to already include back-friendly exercises and habits. One reason for non-results could be the low frequency of school training.

In addition, the study confirmed that this teacher-led back education programme is worthy of inclusion at school. Around 85% of the children received permission from their parents to participate in this study. The programme fit well in a German school year and the dropout rate (until the post-tests at the end of the school term) was low. Results show that the back-pain rate could not be lowered, even with improvement of back-care knowledge, and some back-friendly behaviour hints; research on the effectiveness of the programme should continue.

## Additional file

Additional file 1: Appendix. (DOCX 519 kb)

## Abbreviations

BMI: Body mass index
CG: Control group
IG: Intervention group
