# Supplementary material for: Cluster-randomized, controlled evaluation of a teacher led multi factorial school based back education program for 10 to 12-year old children
Source: BMC Pediatr. 2018 Sep 26;18:312. doi: 10.1186/s12887-018-1280-y (PMC6158865; doi:10.1186/s12887-018-1280-y)
Supplement: Supplementary file 1 — Appendix. (DOCX 519 kb) [file 12887_2018_1280_MOESM1_ESM.docx]

**Additional file 1**

1. Knowledge test
2. Health questionnaire
3. Examples of the posters (mobilisation)
   1. Rolling shoulders
   2. Stretching upwards
4. Examples of the exercises (invigoration)
   1. Crunch (situp)
   2. Lifting the hips
5. **Knowledge test: “Backfit - our school participates”**

THE SPINE

1. How many vertebrae does man have? (cervical, thoracic, lumbar spine)

*Tick ​​the right solution!*

- 15
- 24
- 27
- 30

1. Draw the course of the spine into the male with various activities:

Figure taken from Krämer, J. (1986). Bandscheibenbedingte Erkrankungen. Stuttgart: Thieme, p.268.

1. How does an intervertebral disc live?

- With blood
- At expense via diffusion
- At relief via diffusion
- Best when sitting

1. Why does our spine have a so-called double-S-shape?

*Tick the right solution!*

- So that we can bend and stretch the spine
- The shape of the spine has no special meaning
- To be able to walk upright
- So that shocks can be cushioned

1. How can you improve a bad attitude? *Tick the right solution!*

- Buy a new desk chair
- No longer watching TV on the couch
- With sport and movement
- Doing homework just standing

1. What posture deviations do you know? Which is the optimal posture?

Figure taken from Kendall, F.P., McCreary, E. & Provence, P.G. (2008). Muskeln Funktionen und Tests. München: Urban & Fischer, p.73.

*Assign them to the pictures with the letters A, B, C and D:*

( ) Flat back

( ) Hunchback

( ) Normal back

( ) Hollow round back

1. Why is a round-backed pose harmful?

*Tick the right solution!* **Several answers are correct**

- Unfavorable load on the intervertebral discs
- The spine can stiffen
- Lapping the ligaments on the spine
- Further attenuation of the back muscles
- Shortening of the chest muscles

1. What promotes a hunchback?

*Tick the right solution!* **Several answers are correct**

- Wrong carrying the schoolbag
- Sitting at low tables
- Sitting in high tables
- Wrong diet
- Too weak muscles

LIFTING AND CARRYING

1. Arrange the occurring disc pressures in the following positions:

sit upright - stand - lie - straight raise - crooked raise - sit crooked


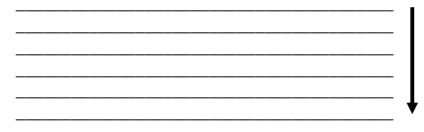
lowest pressure

Highest pressure

1. Name three basic rules for lifting and carrying:

- Bend the knees
- Lift and carry far away from the center of gravity
- Distribute loads evenly
- Wear school bag over one shoulder instead of in hand
- Lift and carry close to the center of gravity
- Let legs stretched
- While wearing the upper body and not the feet turns

SCHOOL BAG

1. How heavy should the schoolbag be at healthy children compared to theri bodyweight at most?

*Tick the right solution!*

- 5 %
- 10 %
- 20 %
- 25 %
- 40 %

SITTING

1. What is the best seating position? *Tick the right solution!*

- Always lean on your back
- Always sit straight
- Switch between different seating positions
- It does not matter how you sit

1. **Health questionnaire**

Name:_____________________ Birth-date.:____________________

Class:____________________

Please mark the correct answer:

1. Does your child suffer from an inborn illness?

- If yes, which one? :_____________________________________
- No

1. Did your child had some injuries?

- If yes, which one? :_____________________________________
- No

1. Did your child receive a surgery?

- If yes, which one?:_____________________________________
- No

1. Has your child been diagnosed with a spine illness?

- Yes
- No

1. Does your child practise sport?

- If yes, which one:_____________________________________

How often:______________________________________

- No sport
- Club sport

1. How many hours does your child is physical active during the day?

- Less than 1 hour
- More than 1 hour
- More than 3 hours

1. Does your child complain about back pain?

- Yes
- 1x per week
- 1x per month
- 1x in 6 montjs
- ______________

If „yes“, when?

- While sitting
- While standing
- During sports
- During long lasting physical activity
- ______________
- No, my child did not suffer from back pain

1. Did your child suffer in the last week of...…

(Please mark what is appropriate)

- Back pain yes no
- Stomache ache yes no
- headache yes no

1. Did your child do the following due to pain…

(Please mark what is appropriate)

- … taking medicine yes no
- … consulting a doctor yes no

1. How does your child judge the following statement?

„I am feeling healthy“

- Never
- Mostly
- Every time

1. How does you child carry the school-bag?

- rolling
- like a shoulder bag
- like a breif case at the side
- like a rucksack very low
- as a rucksack in the higher part of the back
- as a tight back back

1. **Examples of the posters**


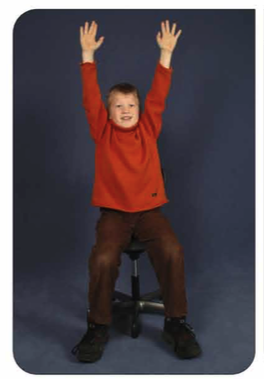

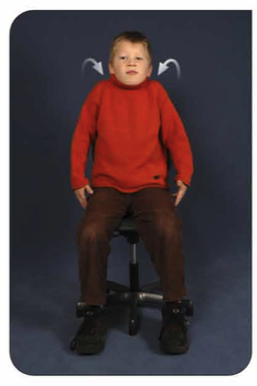


1. **Examples of the exercises**

**
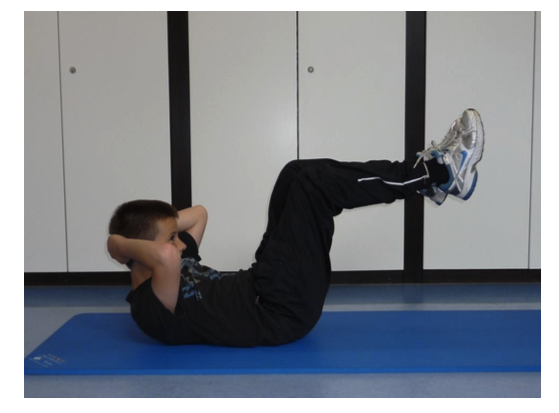
**

**
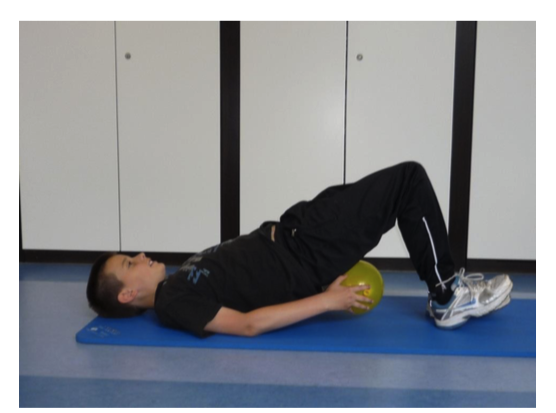
**
